# Comparison of Male Adolescents’ Physical Fitness Using Physical Activity Promotion System and Circuit Exercise Program

**DOI:** 10.3390/ijerph18147519

**Published:** 2021-07-15

**Authors:** Byung-Sun Lee, Seon-Yeong Shin, Yeon-Oh Han

**Affiliations:** 1 Health Physical Activity Institute, Subin Art Inn Building, 25 Eonju-ro 159-gil, Gangnam-gu, Seoul 06024, Korea; shotace@khu.ac.kr (B.-S.L.); healthlab.shin@gmail.com (S.-Y.S.); 2 WEPEAK, Subin Art Inn Building, 25 Eonju-ro 159-gil, Gangnam-gu, Seoul 06024, Korea

**Keywords:** adolescents, physical fitness, circuit exercise program, PAPS, school physical education, physical activity

## Abstract

The purpose of this study is to compare the physical fitness level of adolescents through a physical fitness assessment and a circuit exercise program. A total of 142 middle school students participated. Physical education class consists of a physical fitness assessment, namely, physical activity promotion system (PAPS), and a circuit exercise program. The PAPS measurements include endurance, flexibility, strength, power, body mass index, and total score. The circuit exercise program consists of twist spine, hand walking, rolling squat, cross knee up, jumping and squat, and level-up pacer. First, there were significant differences in PAPS and circuit exercise program according to grade. Second, there was a significant difference in the results of the circuit exercise program according to the level of each physical fitness variable of PAPS. Third, significant correlations were found in the results of the PAPS and circuit exercise program. The use of a circuit exercise program to measure fitness for adolescents can offer convenience for school physical education and be of value as a measure of physical fitness for adolescents. In addition, the circulatory exercise programs used in this study are thought to be applicable to exercise prescriptions to improve endurance, strength, and BMI.

## 1. Introduction

Due to the coronavirus disease (COVID-19) outbreak, social distancing and prohibition of assembly are being imposed worldwide. As a result, the Ministry of Education has proposed postponing school opening, closing school, and adjusting the number of school days as a countermeasure [1]. Accordingly, each school conducted online school opening, sequential school attendance, and non-face-to-face classes, leading to restricted students’ school life and physical activity (PA).

During the adolescent phase in students, numerous changes and development occur physically, mentally, and socially, and proper PA positively affects emotional stability, growth, and development [2]. Hallal et al. (2006) emphasized that adequate PA levels in adolescence positively impact mental and physical health, and health levels in youth are an essential factor in determining health levels after adolescence [3]. Lee (2015) reported that PA in adolescence is crucial for growth and development. Adolescents who continue to participate in PA are taller and have lower body mass index (BMI) than those who do not participate [4].

In WHO (2020) recommendations for PA for adolescents, it is recommended to perform moderate-to-vigorous physical activity (MVPA) for at least 60 min each day, considering the development of adolescents’ physical and mental health and their effects on the whole life [5]. However, Guthold et al. (2020) surveyed the activity of adolescents in major countries with WHO, and four out of five teenagers worldwide were found to have insufficient PA. Notably, 94.2% of Korean adolescents showed a lack of PA [6]. Furthermore, in a study by Jang et al. (2020), MVPA, a recommended exercise intensity for adolescents, was investigated for middle school students. Middle school students’ total PA and MVPA duration appeared the most by the day they had physical education (PE) classes during the semester, followed by days without PE classes during the semester and during vacations. Thus, PE classes at school played a critical role in the PA time of middle school students [7]. 

Since 2009, the school has been implementing the physical activity promotion system (PAPS) as part of school sports to prevent obesity and physical deterioration among Korean students. The PAPS provides information on physical fitness (PF) and health conditions through comprehensive fitness assessments and exercise prescriptions based on PF. PAPS is being implemented to effectively manage students’ PF and establish a system to promote student sports activities [8]. As a follow-up measure of PAPS, efforts are being made to improve student health and PF through PE and after-school special sports activities, especially for students with low PF evaluation. However, there are practical difficulties in inducing active participation of students and guidance of teachers, and the necessity of finding new methods is suggested [9].

Various exercise methods such as aerobic exercise, resistance exercise, and circuit exercise program (CEP) have been used to improve the PF of adolescents. Mainly, CEP can be used in combination of diverse exercises and are effectively known for their positive effect on physique and PF. A study by Han et al. (2000) found that both aerobic exercise and CEP showed similar results in improving body weight and blood lipids, indicating that they are effective in promoting adolescent health [10]. In addition, the CEP performed on obese children improved the functional fitness and PF factors of cardiorespiratory endurance, muscular endurance, power, flexibility, agility, and balance [11]. The results of these prior studies suggest that aerobic exercises, resistance exercises, and CEP, which combines aerobic and resistance exercises, are effective in improving PF and weight control in adolescents. The “Move Challenge” of the Healthy Physical Activity Institute (2020) organized a CEP based on the values of PA presented in the PE curriculum, such as health, challenge, and competition. The CEP consists of six fitness movements and contributes to improving the health and PF of students through PA at varying intensities [12].

As shown above, the PF of adolescents in Korea is very low by worldwide standards, and PAPS is conducted at school PE class to manage PF and exercise prescription of adolescents. However, recently, due to COVID-19, it is difficult to participate in both school PE class and PA to improve the PF of students. Therefore, it is believed that it would be meaningful to check the status of PF levels for middle school students with a CEP that can be carried out on its own without restrictions on locations. The purpose of this study is to confirm the PF level of middle school students with a PF improvement program in the form of CEP and to provide basic data for CEP development for PF improvement.

## 2. Materials and Methods

### 2.1. Subjects

The subjects for this study were 200 selected middle school males aged 14 to 16. A statistical power test was performed by setting an effect size of 0.3 and a power of 0.8 using G*power, and the final 200 subjects were selected in consideration of the 30% omission rate. The records of 142 people (1st grade = 48, 2nd grade = 48, and 3rd grade = 46), excluding 58 people who had insufficient records, were analyzed. We provided a sufficient explanation of the purpose and method to PE teachers. They executed PAPS and CEP as PE classes during the 2020 school year and then supplied measurements except for the students’ personal information such as body weight and height. The physical characteristics of the subjects are shown in Table 1.

### 2.2. Measurements

Subjects were measured for the data of both PAPS and CEP in two separate PE classes during the semester. The two tests had a washout period of one week, and the order of implementation of PAPS and CEP in each school was randomly selected. 

The PAPS consists of five measurements, and each school equally measured and documented the following measurements. The records of PAPS were classified according to each grade criteria table provided by the Ministry of Education. The PAPS criteria are shown in Table 2. The three values were classified as follows (high: Lv 1 and 2, middle: Lv 3, low: Lv 4 and 5) [8]. For the endurance criterion, the ability to run a regular distance (20 m) shuttle run was measured. Students run to the signal sound, while the interval gradually decreased. The number of round trips was measured and recorded as a score. For the flexibility criterion, the degree of bending of the upper body in a sitting position with both feet straight was measured in 0.1 cm increments and recorded as a score. For the strength criterion, the force of gripping the grip meter with four fingers and thumb was measured in 0.1 kg increments and recorded as a score. For the power (standing long jump) criterion, the nearest straight distance from the start point to the landing point was measured. It was measured in 0.1 cm increments and recorded as a score. For body mass index (BMI), students’ height (cm) and weight (kg) were measured. BMI (kg·m^−2^) was calculated, and the score was recorded. The total score was the sum of all five measurement item scores in PAPS. 

**Figure 1:**
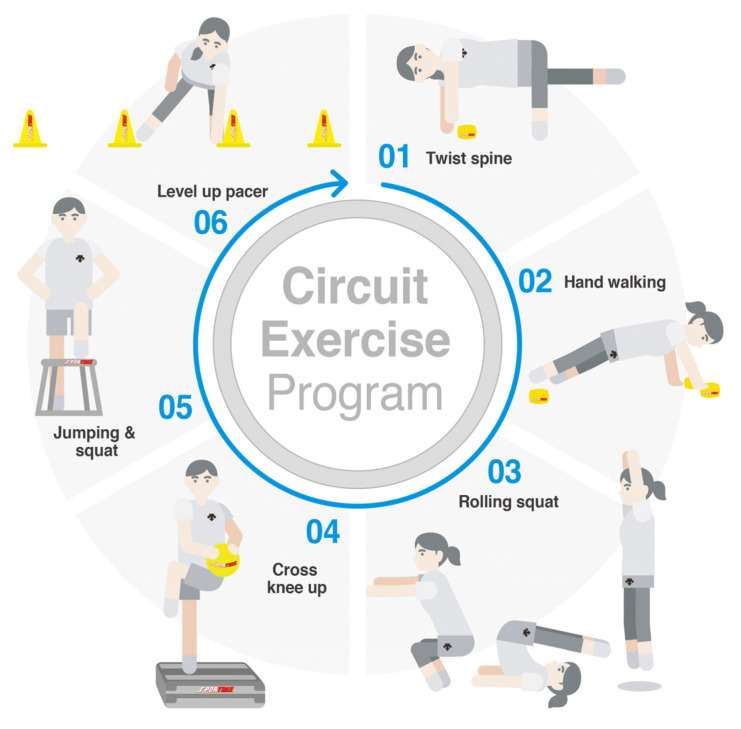
Schematic representation of circuit exercise program.

The CEP in this study utilized youth physical activity promotion programs and consisted of fitness movements to promote health and fitness elements of endurance, flexibility, strength, and power [12]. The CEP consists of six movements, as shown in Figure 1. All actions were performed in sequence and recorded in seconds increments at the end of all movements. Twist spine (flexibility): put one leg over the pelvis, fix it on the floor, and then move one bean bag while turning the upper body to the other side (two sets of three repetitions for each side left and right). Hand walking (strength): in the push-up posture, move one bean bag by hand to the tip of the toe in order and then return to the place (one set of two repetitions). Rolling squat (strength): prepare in a squat position, roll back until toes are over your head, and roll forward to squat position (one set of five repetitions). Cross knee up (endurance): first, cross-jump on the step box (10 repetitions) while holding the ball. Second, bend your knees and cross the ball diagonally (5 repetitions on each side alternately). Third, extend your knees and cross the ball diagonally (5 repetitions on each side alternately). Jumping and squat (power): first, jump on top of the box with both feet (five repetitions). Second, step up the box (5 repetitions). Level-up pacer (endurance): Shuttle run by touching the four cones standing at 1.5 m intervals in sequence.

### 2.3. Statistics

The results from this study were analyzed using SPSS PC^+^ for Windows (version 20.0). The following features were considered: (1) descriptive statistics of all variables are presented as mean (M) and standard deviation (SD); (2) one-way ANOVA was conducted to analyze the differences in the mean between dependent variables for each grade and PF level. If the mean difference in each grade is significant, then a post hoc (*Scheffe* test) was conducted; (3) *Kruskal–Wallis* Test was conducted to analyze the difference in CEP records according to BMI level. If there is a significant difference by BMI level, a *Mann–Whitney* post hoc was performed; (4) to correlate the dependent variables between the two tests, we performed a Spearman correlation coefficient analysis; (5) the significance level (*a*) for all statistical analyses except Mann–Whitney was set to 0.05. The significance level (*a*) for *Mann–Whitney* statistics analysis was set to 0.0167.

## 3. Results

### 3.1. Comparison of PF Levels by Grade

Table 3 shows the results of the comparison of PF levels by grade. There were significant differences between grades in power (*p* = 0.000), BMI (*p* = 0.001), and CEP (*p* = 0.000). However, there were no significant differences between grades in endurance, flexibility, strength, and total score.

### 3.2. Comparison of CEP by PAPS Levels

Table 4 shows the results of analyzing the CEP according to the PF level of the PAPS category. In terms of endurance (*p* = 0.000), flexibility (*p* = 0.001), strength (*p* = 0.001), power (*p* = 0.000), BMI (*p* = 0.001), and total score (*p* = 0.000), there were significantly differences between PAPS levels.

### 3.3. Comparison of CEP by BMI

The results of the CEP were compared by BMI of underweight (1st grade: less than 15.5, 2nd grade: less than 15.9, 3rd grade: less than 16.5), normal weight (1st grade: 15.6~22.9, 2nd grade: 16.0~23.3, 3rd grade: 16.6~23.7), and overweight (1st grade: 23.0 or more, 2nd grade: 23.4 or more, 3rd grade: 23.8 or more), and the results are shown in Table 5. There were significant differences in the three groups (*p* = 0.000).

### 3.4. Correlation between PAPS and CEP

The results of the correlation between PAPS and CEP are shown in Table 6. Significant negative correlations were found in endurance (*r* = −0.809), flexibility (*r* = −0.280), strength (*r* = −0.287), power (*r* = −0.660), and total score (*r* = −0.705). There was a significant positive correlation in BMI (*r* = 0.469).

## 4. Discussion

The physique of adolescents continuously develops and grows, while PF decreases. An increase in physique is due to an improvement in dietary level, while growth in obesity and decrease in PF such as endurance and power is due to lack of exercise [13]. In previous studies, the PF level of a student is an important cause of cardiorespiratory and various causes of death rather than weight status [14]. PF level and PA habits during childhood and adolescence are presented as essential periods for determining PA habits throughout life [15]. School PE provides an important opportunity for PA in childhood and adolescence, and active PA is encouraged [16]. In addition, a study by Jamner et al. (2004) reported that school PE could increase PA in adolescent students and prevent the decrease in cardiorespiratory fitness [17]. Several educational and health institutions provide guidelines for improving adolescent fitness. The US Center for Disease Control and Prevention and the UK Associations for Physical Education recommend 50% MVPA during physical education classes. Prior research showed that the MVPA activity rate among middle and high school students was 40.5%, while the MVPA level decreased as the grade rose to 48.6% for middle school students and 35.9% for high school students. In addition, it was suggested that research is needed to participate in physical education classes actively [18]. The WHO guidelines for recommending youth PA recommend MVPA 5 days a week or more with at least 60 min per day. However, the proportion of Korean adolescents who participated in PA of moderate or higher was at a very low level of 13.9%, which gradually decreases every year [19].

The increase in students’ physiques and the decrease in PF are causing obesity and becoming an important social problem. Therefore, the need to evaluate the fitness level of adolescent students is increasing. In a study by Lee and Oh (2012), adolescent students with high PF showed lower obesity rates than students with low PF. However, obesity rates have been reported to be limited in presenting physical health standards. Therefore, the necessity of a follow-up study to select the standard considering the purpose, target, and social impact of the test was suggested [20]. In this study, PAPS as a method of measuring adolescents’ PF in school PE in Korea and CEP as a PA method in school PE was conducted, and the PF levels of each grade were compared. The power (*p* = 0.000) from PAPS, the 1st grade, was significantly higher than that of the 2nd grade and 3rd grade. In terms of BMI (*p* = 0.01), the 1st grade was significantly lower than the 3rd grade. However, there was no significant difference in endurance, flexibility, strength, and total score. In the CEP (*p* = 0.000), the 1st grade was significantly lower than the 2nd and 3rd grades. In this study, lower grades presented both a high level of power from PAPS and CEP records. The higher the grade, the lower the score for the BMI. In the results of the CEP, a low record means a higher fitness level, and the 1st grade showed a higher level of PF than the 2nd and 3rd grades. These outcomes are interpreted as a result of decreased PF through the physique increased as the grade advanced.

PF is a comprehensive ability and a basic element of PA. However, despite the emphasis on the importance of PF, students have lower PF than their standard physique development [21]. In the preceding study, the PF of middle school students by grade was compared using PAPS. There was a significant difference between grades in cardiorespiratory endurance and muscular strength. Therefore, it was suggested that middle school students need to improve cardiorespiratory endurance as their physique is steadily improving according to grade, while health-related PF is rather decreasing [22]. In the results of this study, the power from PAPS decreased significantly with the increase of grade, but there was no significant difference between grades in cardiorespiratory endurance. However, as the grade increases, the PF record tends to decrease, and the CEP results show a significant decrease according to the grade increase, supporting previous studies.

Although many preceding studies have suggested the important role of PA in adolescence, social issues such as the epidemic of infectious diseases and environments with the absence of school sports, opportunities for PA for students are very scarce. In a previous study by Park et al. (2015), the mobile application was used to provide opportunities for voluntary PA to students for 10 weeks. The PA using the mobile application improved the health and PF of the students and induced the students’ voluntary participation in PA. School PE and existing online learning were based on teacher intervention. Still, previous studies have suggested various possibilities of increasing the PAs through participation and implementation of self-directed PA without teacher intervention [23]. In a study by Han et al. (2000), both aerobic exercise and CEP had positive effects on students’ body composition and PF. It was reported that CEP was particularly effective in increasing lean body mass [10]. In comparing PF levels by grade in this study, significant differences were found according to grade level in PAPS and CEP. PAPS measures five items using different PF evaluation tools and presents the PF level for each measurement according to the results. However, there are limitations in presenting and practicing methods of improving PF by each measurement. The CEP carried out in this study is a combination of aerobic and resistance exercises, which can be expected to have a positive effect on both exercises. Providing CEP consisting of fitness movements for students with low PF levels will provide an opportunity to improve their PF through PA participation. It also can be an easy and simple tool for physical education teachers to use in physical education classes to measure and improve PF for adolescents.

Above all, the physique of students is increasing according to the grade level, but the PF is decreasing. Previous studies point out the problem of the current school education system that focuses on improving intellectual ability rather than improving students’ PF. To increase PA for adolescents, direct and indirect interventions by teachers and the school PE facility were required. In order to compensate for the decrease in PE time, it is necessary to provide students ways to participate in various opportunities and methods of PA.

PF makes it easy to perform daily activities and enjoy leisure time efficiently and effectively. PF is divided into health fitness, such as body composition, and functional fitness that determines sports ability. PF tests using these characteristics are widely used to evaluate the effects of health, motor development, physical training, and exercise propensity of children and adolescents [24]. PAPS, which is being conducted in Korean school PE, evaluates students’ health fitness, and functional fitness in five categories: endurance, flexibility, strength, power, and body composition. In this study, the results of CEP were compared according to the level of the PAPS. In endurance (*p* = 0.000), flexibility (*p* = 0.001), strength (*p* = 0.001), power (*p* = 0.000), BMI (*p* = 0.001), and total score (*p* = 0.000), significant differences were found in the CEP records for each level of PF. The level of each component in PAPS affected the CEP records, and the higher the level for each component of PAPS tended to show higher CEP records. 

Several studies have reported the positive effects on body composition and PF of adolescents from different types of PA conducted in school PE. However, the issues of time, place, and lack of sports equipment are being raised. CEP that combines exercise methods, such as aerobic exercise, resistance exercise, flexibility exercise, and plyometric, has been proposed to improve these issues. In a recent study by Yoon and Moon (2018), significant changes occurred through CEP in body composition such as body weight, body fat, body fat percentage, BMI, and in basic PF such as cardiorespiratory endurance, muscular strength, muscular endurance, and flexibility [25]. A previous study by Shekhawat and Chauhan (2021) reported that adolescents’ participation in the CEP could focus on each muscle in the arms, legs, and torso. If carried out regularly, it effectively improves speed and agility by improving muscular strength [26]. In the study of Lee et al. (2009), a CEP using resistance exercise was shown to be effective in improving body composition, flexibility, muscular strength, and muscular endurance [27]. In addition, a study by Kumar and Kumar (2005) reported that a CEP using plyometric is adequate for power improvement by making it possible to exert maximum muscular strength with a short muscle contraction time [28]. 

Therefore, CEP applied with aerobic exercise, resistance exercise, and plyometric exercise will positively impact PF and body composition. CEP plays an important role in improving diverse fitness items. It is thought to be effective in enhancing the PF of students through a professional and individualized PA without the limitation of time, place, and tools at school and home. It is considered that various forms of CEP development and education are needed to improve PF through voluntary practice by students and solve social problems such as adolescent obesity and PF reduction.

In general, it is known that as the body weight increases, the PF appears to be at a lower level. Although obesity cannot be the standard for setting the PF standard, checking the level of PF based on the obesity level has the meaning of comparing the physique level and the fitness level of adolescents. In the preceding study, the PF level of adolescents according to obesity level resulted that the cardiorespiratory endurance factors were similar to that of obesity. Therefore, it was possible to evaluate cardiorespiratory endurance according to obesity level, and the PF of obese adolescents was generally low. However, the similarity between obesity and strength level was insufficient; thus, a comparison of strength level and obesity level was not appropriate [29]. In this study, to confirm the PF according to the body composition level of adolescents, the records of the CEP were compared with BMI level. The results of the CEP records were significantly lower in the underweight group and the normal weight group than in the overweight group. These results are consistent with previous studies that an inverse relationship between obesity and cardiopulmonary endurance exists: the lower the obesity level is, the higher the PF level is.

PAPS is being implemented to measure, evaluate, and manage adolescents’ PF in Korean school PE. However, there are insufficient methods to improve the PF of students and lower the obesity level using the results of PAPS. Therefore, the CEP used in this study was proposed as a method for weight loss and improvement of fitness levels. In the study of Therefore et al. (2009) [30], CEP is a recommended form of exercise for maximizing the aerobic and anaerobic ability of the body. It effectively regulates body composition and has been reported to improve cardiorespiratory endurance, muscular endurance, muscular strength, flexibility, power, agility, and balance. However, there was no effect on body fat percentage, blood pressure, or resting heart rate. It was reported that the age group of 20–29 had a lower body fat percentage and a higher level of autonomic nervous system balance, compared to other age groups, and therefore, there was no significant difference. Another study conducted a CEP in school PE to enhance students’ physical fitness. The results were shown to improve muscular strength, cardiorespiratory endurance, lean mass, and body composition, but there were no changes in blood lipid and C-reactive protein levels [31]. In the study of Mayorga-Vega et al. (2013), it was possible to improve the strength and PF of students through a CEP using elastic bands, balls, and body weight at school sports sites without strength training equipment. In addition, the CEP is recommended as a practical exercise method in school PE because it is possible to participate in numerous types of exercise with minimal exercise time [32].

Obesity and physical deterioration of students are emerging as social problems, and as in this study, results presented that the higher the obesity level is, the lower the PF is. Several ways to prevent and manage these problems are proposed; previous studies have reported positive effects of CEP on students’ PF and body composition. The CEP used in this study was developed to increase the PA for students in various environments. In the field of school PE and PA, it is considered that participation in CEP can be utilized as a program for improving and managing students’ PF and physique. 

PA has a positive effect on the physical and mental development of adolescents. In the global adolescent PA survey conducted by the WHO in 2020, Korean adolescents reported the lowest PA level. Providing varied methods for increasing the amount of adolescent’s PA and opportunities to participate in PA is drawing attention as a crucial task [6]. The Ministry of Education (2009) has distributed and operated PAPS, a health and fitness enhancement program, to improve PA participation opportunities and PF of adolescents, which are constantly decreasing. PAPS supplemented the problems of past student physical ability tests. It is used in school PE fields to diagnose students’ health and PF level through accurate measurement and to provide exercise prescriptions based on the measurement results [8]. In addition, Ko et al. (2005) reported that adolescents’ PA decreased due to social changes caused by economic development and that PF and health problems appeared. To improve the situation of reduced adolescents’ PA and PF, the health promotion program “Adolescents PF Test” was developed. These various preceding youth PF measurement and improvement programs include evaluating cardiorespiratory endurance, muscular strength and muscular endurance, flexibility, agility, and body composition [33]. It is recommended to evaluate students’ health and PF through measurement and establish and operate a health and PF class for students who received a low evaluation.

However, many problems have been raised to teachers and students who are directly operating and using school PE. In the study of Kang et al. (2013), students were not interested in subjects and activities that were not related to grading and were negative about PAPS measurement [2]. Field difficulties for PE teachers in measuring and managing PAPS were excessive work, methods of measuring, evaluating student health and PF, and the hassle of inputting measurement data into the National Education Information System. In this study, correlation analysis was conducted between the PF measurement results of PAPS and the records of the CEP. As a result of correlation analysis, a negative correlation was high in endurance (*r* = −0.809) and the total score (*r* = −0.705). There were significant correlations in flexibility (*r* = −0.280), strength (*r* = −0.287), power (*r* = −0.660), and BMI (*r* = 0.469).

The CEP conducted in this study consisted of six exercises. It provided teachers with ease of measurement and management, while students with an interest in PF and health improvement. CEP is known to be an effective program used not only for the general public but also for elite athletes to increase their PF. Williams and Cash (2001) found that, in contrast to aerobic exercise, CEP consisting of resistance exercise increased the subjects’ upper and lower body strengths. In addition, the results of an assessment of appearance showed increased physical satisfaction, improved physical self-efficacy, and reduced social physique anxiety. Therefore, it was reported that the positive effects of physical and mental factors through CEP were shown as improving strength, cardiorespiratory endurance, and emotional stability [34]. Traditional resistance training methods are effective in improving muscular strength and endurance in a PE environment. However, Henry et al. (2006) suggested CEP because resistance exercise was difficult to adapt individually and evaluate achievement. The CEP is easier for students to check the effect of the resistance exercise. The CEP resulted in increased $\dot{V}$O_2max_ and muscular strength and decreased body fat percentage, effectively improving PF and body composition. The CEP was recommended as a form of exercise suitable for a PE class environment because it provided a high exercise effect. The exercise time was shorter than that of general aerobic exercise [35]. 

In this study, a statistically significant correlation was found in endurance, power, and total score measurements as a result of the correlation analysis between the PF measurements of PAPS and the CEP records in students. Therefore, improvements in endurance and power can be expected through continuous CEP implementation. Thus, the implemented CEP will provide PE teachers with convenience in measuring and managing students’ PF. Additionally, it will provide motivation and interest in students’ participation in PA. In school PE, the exercise effect for a short period of time is high, which is thought to improve students’ physique, PF, and emotionality. In the future, the development and dissemination of various forms of CEP are expected to improve the fitness of adolescents.

## 5. Conclusions

The purpose of this study was to compare the PF level of middle school students using PAPS and CEP. First, there were significant differences in the level of PF for each grade in power, body composition, and CEP records. Second, as a result of comparing the results of the CEP according to the PAPS fitness level, significant differences were found in endurance, flexibility, strength, power, body composition, and total score. Third, there was a significant difference in comparing the results of the CEP according to the BMI level. Fourth, in the correlation analysis between the PF measurement results of PAPS and the results of the CEP, significant correlations were found in endurance, flexibility, strength, power, body composition, and total score. 

In conclusion, the amount of PA for adolescent students is low, and the PF level decreases as the grade advances. In school PE, methods for measuring and managing the PF of students need to be improved, and diverse opportunities and strategies for PAs of students are required. The CEP will provide the convenience of teachers in school PE and contribute to the development of endurance and power through the voluntary participation of students in PA. In this study, the improvement of youth PF through CEP was not confirmed, and there was a limit on the use of secondary data and recruitment of sample groups. In the future, research is needed to confirm the effectiveness of the development and training of various combinations of CEP for adolescents.

Additionally, the “Move Challenge” CEP contains the value of PA of health, challenge, and competition in the PE process. Numerous PAs of CEP are organized to improve health and PF. Through the CEP, we expect the development of the physique and PF through the convenience of PE teachers and the voluntary participation of students in PA.

## Figures and Tables

**Table 1:** Characteristic of participants (Mean ± SD).

Variables	1st Grade (*n* = 48)	2nd Grade (*n* = 48)	3rd Grade (*n* = 46)
Ages (years)	14.00 ± 0.00	15.00 ± 0.00	16.00 ± 0.00
BMI (kg·m^−2^)	19.89 ± 5.22	21.70 ± 4.47	23.88 ± 5.03

BMI: body mass index.

**Table 2:** PAPS criteria.

Variables	Value	Lv 5 (Very Low)	Lv 4 (Low)	Lv 3 (Average)	Lv 2 (High)	Lv 1 (Very High)
score		0~3	4~7	8~11	12~15	16~20
Endurance (repetition)	1st grade	15~19	20~35	36~49	50~63	64~73
	2nd grade	15~21	22~37	38~51	52~65	66~75
	3rd grade	16~23	24~39	40~53	54~67	68~79
Flexibility (cm)	1st grade	−5.1~−4.1	−4.0~1.9	2.0~5.9	6.0~9.9	10.0~25.0
	2nd grade	−5.1~−4.1	−4.0~1.9	2.0~6.9	7.0~9.9	10.0~25.0
	3rd grade	−5.1~−3.1	−3.0 ~2.5	2.6~6.9	7.0 ~9.9	10.0~25.0
Strength (kg)	1st grade	14.4~16.4	16.5~22.4	22.5~29.9	30.0~41.9	42.0~46.0
	2nd grade	19.1~21.9	22.0~28.4	28.5~36.9	37.0~44.4	44.5~47.0
	3rd grade	19.1~24.9	25.0~32.9	33.0~40.4	40.5~48.4	48.5~50.0
Power (cm)	1st grade	122.9~131	131.1~159	159.1~177	177.1~211	211~219.7
	2nd grade	129.9~136	136.1~169	169.1~187	187.1~218	218.1~229.3
	3rd grade	1414~145	145.1~180	180.1~201	201.1~238	238.1~244.0
BMI (kg·m^−2^)	1st grade	~13.3 25~	13.4~15.3 23.3~24.9	15.4~17.3 22.4~23.2	17.4~18.3 21.4~22.3	18.4~21.3
	2nd grade	~13.3 25~	13.8~15.7 23.9~24.9	15.8~17.7 22.9~23.8	17.8~18.7 21.9~22.8	18.8~21.8
	3rd grade	~14.2 25~	14.3~16.2 24.4~24.9	16.3~18.2 23.4~24.3	18.3~19.2 22.4~23.3	19.3~22.3
Total score		0~19	20~39	40~59	60~79	80~100

**Table 3:** Comparison of PF levels by grade (Mean ± SD).

Variables	1st Grade (*n* = 48)	2nd Grade (*n* = 48)	3rd Grade (*n* = 46)	*F*(df)	*p*	*Scheffe*
Endurance	13.67 ± 5.62	13.85 ± 4.52	11.78 ± 4.99	2.3869(2)	0.096	
Flexibility	13.65 ± 4.42	13.77 ± 5.55	14.65 ± 3.68	0.656(2)	0.521	
Strength	11.06 ± 3.56	10.50 ± 5.32	14.91 ± 15.39	2.975(2)	0.054	
Power	16.00 ± 4.41	12.31 ± 4.64	10.96 ± 4.50	15.799(2)	0.000 ***	a > b,c
BMI (kg·m^−2^)	20.27 ± 4.33	21.70 ± 4.47	23.88 ± 5.03	7.286(2)	0.001 ***	a < c
Total Score	64.48 ± 14.94	61.94 ± 16.21	62.30 ± 22.41	0.276(2)	0.759	
CEP	123.69 ± 42.13	152.79 ± 33.11	168.72 ± 56.08	12.372(2)	0.000 ***	a < b,c

BMI: body mass index, CEP: circuit exercise program, a: 1st grade, b: 2nd grade, c: 3rd grade, ***: *p* < 0.001.

**Table 4:** Comparison of CEP by PAPS levels (Mean ± SD).

Variables	*n*	Value	CEP (s)	*F*(df)	*p*	*Scheffe*
Endurance	82	High	124.89 ± 36.98	39.161(2)	0.000 ***	a < b < c
	35	Middle	167.54 ± 36.52			
	25	Low	197.08 ± 46.64			
Flexibility	100	High	138.37 ± 38.94	7.643(2)	0.001 ***	a < b
	26	Middle	172.92 ± 55.16			
	16	Low	168.69 ± 66.94			
Strength	65	High	132.77 ± 28.72	7.959(2)	0.001 ***	a < b
	52	Middle	166.79 ± 63.76			
	25	Low	149.16 ± 36.58			
Power	92	High	130.43 ± 37.25	23.769(2)	0.000 ***	a < b,c
	28	Middle	176.04 ± 45.75			
	22	Low	186.50 ± 53.43			
BMI	59	High	134.12 ± 34.03	10.196(2)	0.000 ***	a,b < c
	27	Middle	134.67 ± 35.32			
	56	Low	169.34 ± 57.90			
Total score	74	High	120.47 ± 28.62	52.495(2)	0.000 ***	a < b < c
	52	Middle	168.19 ± 36.11			
	16	Low	210.69 ± 62.85			

BMI: body mass index, CEP: circuit exercise program, a: high group of PF level. b: middle group of PF level. c: low group of PF level. ***: *p* < 0.001.

**Table 5:** Comparison of CEP by BMI.

Variables	Underweight (*n* = 8)	Normal Weight (*n* = 88)	Overweight (*n* = 46)	*F*(df)	*p*	*Mann–Whitney*
CEP	117.13 ± 38.78	132.23 ± 34.26	189.89 ± 52.56	36.421(2)	0.000 ***	a,b < c

BMI: body mass index, CEP: circuit exercise program, a: underweight, b: normal weight, c: overweight, ***: *p* < 0.001.

**Table 6:** Correlation between PAPS and CEP.

Variables	Circuit Exercise Program		
	*r*	*p*	
Endurance	−0.809	0.000	***
Flexibility	−0.280	0.001	***
Strength	−0.287	0.001	***
Power	−0.660	0.000	***
BMI	0.465	0.000	***
Total score	−0.705	0.000	***

BMI: body mass index, ***: *p* < 0.001.

## Data Availability

The data presented in this study are available on request from the corresponding author.
